# A Qualitative Exploration of the Views of Policymakers and Policy Advisors on the Impact of Mental Health Stigma on the Development and Implementation of Mental Health Policy in Singapore

**DOI:** 10.1007/s10488-021-01171-1

**Published:** 2021-09-29

**Authors:** Mythily Subramaniam, Shazana Shahwan, Chong Min Janrius Goh, Gregory Tee Hng Tan, Wei Jie Ong, Siow Ann Chong

**Affiliations:** 1 Research Division, Institute of Mental Health, Buangkok Green Medical Park, 10 Buangkok View, Singapore, 539747 Singapore; 2 Saw Swee Hock School of Public Health, National University of Singapore, Singapore, 117549 Singapore

**Keywords:** Mental health conditions, Policymakers, Stigma, Barriers to treatment

## Abstract

Few studies have examined the views of policy makers regarding the impact of mental health stigma on the development and implementation of mental health policies. This study aimed to address this knowledge gap by exploring policymakers’ and policy advisors’ perspectives regarding the impact of mental health stigma on the development and implementation of mental health programmes, strategies, and services in Singapore. In all 13 participants were recruited for the study comprising practicing policymakers, senior staff of organisations involved in implementing the various mental health programmes, and policy advisors. Data was collected through semi-structured interviews, which were transcribed verbatim and analysed using reflexive thematic analysis. Data analysis revealed three superordinate themes related to challenges experienced by the policymakers/advisors when dealing with mental health policy and implementation of programmes. These themes included stigma as a barrier to mental health treatment, community-level barriers to mental health recovery, and mental health being a neglected need. Policymakers/advisors demonstrated an in-depth and nuanced understanding of the barriers (consequent to stigma) to mental healthcare delivery and access. Policymakers/advisors were able to associate the themes related to the stigma towards mental illness with help-seeking barriers based on personal experiences, knowledge, and insight gained through the implementation of mental health programmes and initiatives.

**Supplementary Information:**

The online version contains supplementary material available at 10.1007/s10488-021-01171-1.

## Introduction

More than a billion people were estimated to suffer from mental and addictive disorders in 2016, contributing to 19% of all years lived with disability (Rehm & Shield, 2019). Furthermore, research suggests that the cost of mental illness was US$ 2.5 trillion in 2010 and is expected to rise to US$ 6.0 trillion by 2030. This economic burden far exceeds the cost of illness of cancer, cardiovascular disease, chronic respiratory disease, and diabetes (Bloom et al., 2011). Mental disorders are thus a serious public health concern given their high prevalence, early age of onset, chronic course, associated comorbidities, loss of productivity, treatment costs, and increased mortality.

Despite the significant impact of mental illnesses on the individual, healthcare systems, and society, mental illnesses often remain untreated (Subramaniam et al., 2020a). Consequences of untreated mental illness include poor response to therapy, poor quality of life, suicide, and homelessness (Altamura et al., 2010; Diego-Adeliño et al., 2010; Ran et al., 2018). Various explanations have been suggested as to why people do not receive treatment; however, the most widely cited reasons are stigma and the inability to access mental healthcare. Studies indicate that many countries have no or limited budget allocated for mental health (World Health Organization, 2011). At the same time, most countries have limited legislation on mental healthcare policies (Rathod et al., 2017). A recent systematic review by Carbonell et al. (2020) identified several barriers in mental healthcare systems. Notable among these were (i) structural barriers that included weak planning, underfunding, and low prioritization of mental healthcare policies, (ii) health culture, which included stigma and poor mental health literacy, and (iii) rehabilitation management that encompasses lack of human resources and community mental health services. The authors also identified the cost of services and reliance on psychiatric institutions as main service providers as some of the barriers to accessing mental health services.

However, Carbonell et al. (2020) and other authors acknowledge that mental health stigma and the lack of mental health policies are related to each other. While on the one hand, policies are needed to address mental health stigma, on the other, stigma can also influence mental health policies. Research suggests that stigma associated with mental illness acts as a limiting factor for developing policies that would enhance the care and support of people with mental disorders (Corrigan et al., 2004; Purtle et al., 2018). Equity in health has been defined as ‘the absence of systematic disparities in health between groups with different levels of underlying social advantage/disadvantage’ (Braveman & Gruskin, 2003). However, there is much inequity in healthcare, where mental disorders are often neglected in policies and global strategies to prevent and control diseases (World Health Organization, 2012). Changes in population demographics and improved healthcare delivery have resulted in a shift of burden of disease from communicable to non-communicable diseases (NCDs) globally (GBD, 2017 Causes of Death Collaborators, 2018; GBD, 2015 Mortality & Causes of Death Collaborators, 2016). However, mental disorders are not considered NCDs despite the high prevalence of comorbidity between these two groups of disorders, which is associated with poorer health outcomes (Ivbijaro, 2011; Pryor et al., 2017). It was only in 2015 that mental health was finally included in the United Nations (UN) Sustainable Development Goals (SDGs) (Votruba & Thornicroft, 2016). It was then that the UN acknowledged the burden of disease of mental illness, and included prevention and treatment of mental disorders as well as promotion of mental wellbeing as part of the SDGs.

Singapore is a city-state in Southeast Asia with a population of 5.7 million residents comprising Chinese, Malay, Indian, and Other ethnic groups. Mental health care in Singapore is provided both in the hospitals and at the community level. The Institute of Mental Health is the state mental health hospital comprising about 1800 in-patient beds and an out-patient caseload of 47,000 patients in a year. Other public and private hospitals also provide in-patient and out-patient mental health care but in smaller capacities. Mental health care in the community is provided by primary care physicians working either in polyclinics or as General Practitioners (GPs) as well as psychologists and counselors working in voluntary welfare organisations (Peh et al., 2021).

In 2006, Singapore launched its first National Mental Health Blueprint (NMHB) for the promotion of mental health and to reduce the incidence and impact of mental health problems. A key component of the blueprint was a shift from an institutional model to a community-based one. Subsequently, the Community Mental Health (CMH) Masterplan was launched in 2012 to further strengthen the care of people with mental disorders and dementia in the community (Ong, 2017). As with most developed countries, there are laws in Singapore that safeguard the interest and safety of people with mental health conditions in Singapore. These laws include the (i) Singapore Mental Health Care and Treatment Act which provides for the involuntary admission of a person with mental illness for care and treatment; and the (ii) Mental Capacity Act which allows a person older than 21 years of age to make an advance medical directive or a lasting power of attorney (Ho et al., 2015). As part of the community-based sentences introduced in 2010, community courts can also issue a Mandatory Treatment Order (MTO) and direct an offender suffering from certain treatable psychiatric conditions to undergo psychiatric treatment for a period of no longer than 24 months (Republic of Singapore, 2010). In addition, the Vulnerable Adults Act (vulnerable adult is defined as an individual aged 18 years and above, with mental or physical disabilities, and is unable to protect himself or herself from abuse, neglect, or self-neglect) came into operation in 2018 in Singapore. This act seeks to safeguard vulnerable adults from abuse, neglect, or self-neglect (Republic of Singapore, 2018).

Despite policymakers’ significant and active role in determining the services for mental disorders, few studies have examined policymakers’ views regarding the impact of mental health stigma on the development and implementation of mental health policies. For the purpose of this study, we defined mental health policy as “an organized set of values, principles, and objectives for improving mental health and reducing the burden of mental disorders in a population.” (p12, World Health Organization, 2005) which is often implemented as plans, programmes, strategies, and legislation. This study aimed to address this knowledge gap by exploring policymakers’ and policy advisors’ perspectives regarding the impact of mental health stigma on developing and implementing mental health programmes, strategies, and services in Singapore.

## Methods

This study is part of a larger research project that explores the concept of mental illness stigma among different stakeholders, including the public, persons with mental illness (PMI), caregivers of PMI, healthcare professionals, and policymakers/advisors in Singapore (Ong et al., 2020; Tan et al., 2020).

### Participants and Setting

The demographics of the participants are provided with limited details to protect their confidentiality. In all, 13 participants were recruited for the study from Mar 2019 to Oct 2019. The participants included seven females and six males. Their ages ranged from 42 to 65 years. Of these, 10 participants were practicing policymakers in the various ministries in Singapore and senior staff of organisations involved in the implementation of the various mental health programmes. In addition, three participants were identified as policy advisors whose role was more advisory, e.g. to inform the policymakers on the need for various programmes and services or provide feedback to them as part of advisory committees. Participants were recruited by either direct email invitation seeking their participation or via snowball sampling. Inclusion criteria included being actively involved in mental health policy formulation or implementation and willingness to partake in a face-to-face interview, which would be audio recorded.

Prior to initiating the data collection process, the Institutional Review Board (National Healthcare Group Domain Specific Review Board) reviewed and approved the study. Before each interview, the interviewer explained the purpose of the study, the informed consent process, and confidentiality. All participants gave written informed consent for participating in the study.

### Data Collection

Data collection was done via semi-structured interviews (SSIs). All SSIs were conducted by either MS or SS and lasted between 60 and 90 min. SSIs were done either at the place of work or other venues preferred by the participant. At the start of the session, background information (i.e., age, gender, education level, ethnicity) was collected from the participants using a simple socio-demographic form. An interviewer conducted each SSI with a note taker present. The two interviewers were trained and experienced qualitative researchers. The topic guide that was developed by the study team was used for all the SSIs (enclosed as an appendix). The SSIs were audiotaped and transcribed verbatim for analysis.

### Analysis

The data were analyzed using reflexive thematic analysis (Braun et al., 2019), which involves identifying patterns through data familiarisation, data coding, and theme development and revision. The transcripts were distributed between the two study team members conducting the interviews (MS and SS), who independently identified preliminary codes from their respective transcripts. This was followed by the generation of initial themes, which were then further refined. The themes were then defined and named by the coders. Regular research meetings were held throughout the coding process to allow discussions. Brief notes were recorded as a means of establishing an audit trail and for keeping track of emerging impressions of what the data meant and how they related to each other. After each meeting, the two coders documented any changes to the themes (combining/ discarding or refining) clearly in the codebook. In order to ensure consistency of coding, the same transcript was coded by both coders to establish inter-rater reliability. Finally, the two coders discussed and repeated the coding with another transcript until a satisfactory inter-rater reliability score was established (Cohen’s kappa of 0.70). Data analysis was conducted with Nvivo Version 11.0.

## Results

The data analysis revealed three superordinate themes, which were related to challenges experienced by the policymakers/advisors when dealing with mental health policy and implementation of programmes. These themes included stigma as a barrier to mental health treatment, community-level barriers to mental health recovery, and mental health being a neglected need. The superordinate themes comprised themes and subthemes, which have been described and are supported by minimally edited verbatim quotes from the participants. Policymakers/ advisors also discussed what they considered were facilitators of recovery and provided suggestions that could facilitate recovery, which emerged as the final superordinate theme and is not addressed in the current article. Two themes comprising ‘culture and mindset of Singaporeans’ and ‘mental health a poorly understood condition’ overlapped significantly with the themes that emerged from the sub-study exploring public attitudes towards stigma (Tan et al., 2020) and hence have been discussed briefly in the current article.

### Stigma as a Barrier to Mental Health Treatment

Policymakers/advisors unanimously acknowledged that stigma played a significant role in the treatment gap, as observed in local studies. However, they opined that the provision of services alone might not help bridge this gap. In their view, there were several ways in which stigma could deter someone from seeking help. The themes that emerged from their verbatims included:

*Avoidance of labeling and fear of disclosure*– Policymakers/advisors were unanimous in their view that despite having multiple options of help, most of which were accessible and affordable, people were not willing to seek help for their mental health conditions. They attributed it to the fear that it could adversely affect their job, relationships, and their life as a whole. Most policymakers/advisors spoke anecdotally of community workers who would identify and refer someone with mental illness to formal care. But they would typically be unsuccessful in convincing the person due to concerns about a diagnosis on their medical records, which could lead to disclosure (potentially). A few mentioned that the Employee Assistance Programmes (EAP) in Singapore were not successful as they were associated with the place of work and hence led to the concern that the EAP provider would convey sensitive information to the employer despite assurances of confidentiality. These were reflected in verbatims such as.

*“Because they’re afraid how that would affect their job, their family. So, I mean we have also seen some of the people we spoke to, some of our cases that we have to deal with; then they’ll ask, oh does that mean I go and see...? Even parents say, “does that mean I’ll go and see the doctors? Who will have the record? I don’t want to have that record there.”*

*“Then we talk about confidentiality - ‘when I send you for counselling, or you go yourself, I would never know. All I need to know from that company if I pay them is to tell me the aggregate number.’ But you know, straight away, everybody gets very nervous. In Singapore, the EAP doesn’t work well because nobody wants to go. They’re very afraid.”*

*Anticipation of stereotyping and discrimination- The majority of the* policymakers/advisors felt that a person might want to avoid being stereotyped. Those with mental illness may be perceived as lazy or weak and therefore unable to perform or shoulder responsibilities or be seen as not productive enough. A couple of policymakers/advisors agreed that productivity was important in Singapore’s context. Therefore, when a person was symptomatic, they would not be able to function fully at work, making things difficult for the organization and co-workers and leading to interpersonal tensions. To avoid such workplace-related discrimination, they felt people would neither declare nor seek treatment for mental health conditions.

*“And that’s why the productivity part of it is important, but mental health conditions affect productivity. So definitely, no matter what, it affects your productivity, and that’s…that is something that will become problematic and that’s why people don’t want to come forward, especially depression because it affects them, and people’s view of their ability to work.”*

*Lack of mental health literacy and normalisation of symptoms by young people- The majority of* policymakers felt that schools were still not discussing mental health conditions openly enough. They felt that mental health literacy was only lightly introduced in schools as part of a broad topic on socio-emotional health as this was more acceptable to parents. However, the inadequate coverage resulted in poor knowledge of mental health conditions and sources of help-seeking. While they were of the opinion that emphasis on suicides might be associated with copycat cases, they still felt a need to educate students and have open discussions with them. The participants felt that mental health problems like eating disorders and self-harm, common in young people, might get normalized if they were not addressed openly and carefully. Young people would neither seek help nor alert a responsible adult if they realized their peer was doing that.

*“And they say yea we have the social and emotional learning framework (SEL) because we have the social, emotional competency. So I said, okay, why can’t you call it mental health? And they said no because you know, it is not… it’s just the language. So I asked them, ‘Why.’ Because I know that SEL has been around for a while, right. And I know the intent is good; it is well-intended. But when I check with young friends, teachers, I will be frank with you. I have very many teacher friends right, and they do not see an immediate link between SEL and mental health.”*

*“So, I mean that’s why I said like self-harm and eating disorders to a certain degree have been normalized, they don’t see it as a mental disorder or see a need for help, you know. So… things like that, in their minds, a mental illness or a mental health condition probably has a very different meaning.”*

*Family stigma –* Policymakers/advisors were unanimous in saying that parents found it especially hard to accept that their children had a mental health condition and they delayed seeking treatment. They also attributed it to a lack of mental health literacy, which led to parents making decisions that would not benefit the child in the long term. Participants also felt that family members may pressurize children and prevent them from disclosing their symptoms to others, leading to delays in seeking treatment. Due to stigma, families may also be embarrassed, or lack empathy towards a person with mental health conditions and end up being unsupportive of the person with mental health conditions.

*“I mean it’s… and because also the thing with the younger ones is that they don’t know where to go for help, right? Especially if they think that their parents think that they’re just, you know…. yea, coming up with something that they don’t believe and all that, and then they really don’t have any place to go for help.”*

*Structural stigma –* Structural stigma for this study was defined as a ‘social process that represents institutional policies that intentionally or unintentionally restrict opportunities and results in health disparities among people with mental illness (Campbell & Deacon, 2006; Corrigan et al., 2004). The majority of the participants brought up several aspects of healthcare delivery and other organizational regulations in Singapore that may unknowingly act as deterrents to help-seeking. These included providing specialist mental health services through a tertiary mental health provider rather than as part of primary care services. Participants also felt that the notion of having a separate hospital or organization that exclusively provided services to people with mental health issues ensured cost-effectiveness and efficiency but impeded the development of community mental health initiatives. Moreover, despite being staffed by specialists and services that were the best in the field, a facility devoted to mental health services would be associated with stigma, which inadvertently deterred people from seeking help. On the other hand, the majority of the participants were also cognizant that long-term care institutions were not keen to admit a person with mental health conditions and expected them to seek help at the tertiary care hospital. Another concern expressed unanimously by participants was the need to declare mental health conditions either when applying for a job or enlisting for National Service (NS), which resulted in label-avoidance and lack of help-seeking. Some participants pointed out that getting insurance for a person with a mental health condition was not easy in Singapore. Given the early age of onset of mental disorders and the high risk of comorbidity with physical illnesses, many participants felt that this was an area that needed to be addressed. Failure to do so would result in parents or persons with mental illness avoiding a diagnosis or not revealing to their healthcare provider (non-mental health care professional) about their mental health condition.

*“And where I’ve been asked for help, I’ve been asked to suggest or recommend referrals; I get the answer. “No, my wife just won’t, will not accept that fact for my son to go to XX hospital”. And that’s not that long ago. It’s sad and saddens me because I feel that the parent is denying healthcare or support in a way.”*

*“I mean for example, army (NS), they have to tell the army that they have been seen at the child and adolescent mental health services. So I mean, I know these are concerns. I understand you have to balance it with… you have to balance it with the safety of others and all that, but it’s…”*

*“I think insurance comes up very, very often whenever I have a dialogue, whenever I have a focused discussion, persons with recovery lament about, you know, they can’t get insurance. Their families and caregivers tell us they can’t get insurance, she’s so young, he’s still so young, what are we to do.”*

### Community-level Barriers to Recovery of People with Mental Health Conditions

For the purposes of this theme, policymakers/advisors defined community broadly as a group with commonalities in terms of culture, norms, values, and religion, who share a sense of purpose and belonging. However, the shared culture, values, and norms lead to the marginalization of those perceived as different. In the context of this theme, policymakers/advisors expressed their challenges in creating inclusive communities that would accept people with mental health conditions.

*Culture and mindset of Singaporeans* – All the policymakers felt that the Singapore society has a structure that reflects the culture, history, and philosophy of the people, where there is a lot of emphases placed on efficiency, effectiveness, and productivity. Participants were also of the opinion that most of the time, communities only notice the few cases of PMI with poor outcomes (despite best efforts of community and healthcare workers) as they stand out, which leads to the strengthening of the negative stereotypes. Participants also felt that a prerequisite for integrating persons with mental health conditions was a supportive and empathetic community. When such an environment was lacking, institutional care would become the preferred choice over integration as it would at least ensure shelter and care for the PMI.

*“But then you have those who would be able to function outside (an institution). But to be able to function outside, they must have some sort of again, I talk about this ecosystem, where you can have that supportive environment where people recognize, understand the challenges that come with those conditions.”*

*Not in My Backyard (NIMBY)* – The majority of the policymakers also candidly acknowledged that the desire to keep people with mental health conditions away from community spaces which included primary healthcare clinics, and reluctance to build daycare centres for people with mental health conditions in their neighbourhood stemmed from their fear of violent behavior. Policymakers felt that communities equated mental illnesses with violence and aggression and thus preferred to distance themselves.

*“Though having said that, years ago, this was when XX Centre just started. I went to a daycare centre in the void deck, and I was going to visit, and the centre manager said “when you come, make sure…” – and she scared the daylights out of me – “don’t wear any jewellery, don’t wear anything, they’ll pull, don’t make eye contact and just be very watchful.”*

*Mental Illness Remains a Poorly Understood Condition* – The majority of respondents felt that the general public did not understand why a person with mental illness was unable to perform optimally, which led to them being labelled as lazy or difficult. This could be both in terms of activities of daily living such as bathing and grooming as well as role functioning – e.g. as a spouse or father, unlike a physical disability that is apparent and gains a more empathetic response from the community. Other participants felt that the causes of mental illnesses remain poorly understood, and people still attributed mental illness to supernatural causes or even as a punishment for their past deeds.

*“But when you look at mental illness, it’s very difficult to see, and because of that, it makes it difficult for people to empathize, so sometimes that may be contributing to the stigma. I haven’t figured out how; I wonder how people always feel that yeah I look at a person in a wheelchair, I tend to like agree that this person needs help, but when I see someone with mental illness or person with depression, for example, I always label it as lazy.”*

*Lack of Sustained Anti-stigma Efforts* – All the policymakers/advisors, felt that anti-stigma efforts in Singapore were lagging behind other countries. For example, policymakers/advisors cited campaigns from the UK that had been well-funded and had a significant reach while in Singapore, most campaigns were of a much smaller scale. Policymakers/advisors were also cognizant of the need to demonstrate the outcomes of such campaigns as they were unsure whether they could result in significant and lasting change.

*“In other countries like in the UK, when I saw some of the messaging that they put out there, I realized that they had been more open with these issues a long time ago. So maybe the issues are the same, but the prevention efforts that have been taken started many years ago.”*

### Mental Health as a Neglected Need

Policymakers mentioned three themes: lack of resources and budget for mental health, mental health not being a priority (in schools and workplaces), and forcing mental health into a ‘health box.’

*Lack of resources and budget for mental health* – The majority of the policymakers/advisors felt that mental health was being given more attention and resources in the past decade as compared to the years before that. However, half of them pointed out that mental health conditions were not given the same importance as physical health conditions even though they were equally or even more prevalent. The majority also stated that resources were still limited to hospital services, while mental health conditions had to be treated in the community. Participants also remarked that home care services needed to be funded as people with severe mental health conditions would otherwise be unable to access care.

*“No, let me rephrase that. Less difficult. Far less difficult to get support in this area than in the past (referring to funding).”*

*“Yes. That is why, you know, I wanted to objectively let them know 1 in 4 for ageing, right, 1 in 9 for diabetes, 1 in 7 for mental health. So, you know you think about the resources given to these as priorities, this (mental illness) also is as much of a threat to… I mean if you talk about the economy and the society, I mean this has a similar impact."*

*“In other countries, they do in-home behavioural support, whereas we hardly have a chance to go in-home psychologists. We don’t have enough psychologists to do in-home work. So then you send somebody else who doesn’t know what they are doing, they just go and ‘chat chat’… go out, then there’s no intervention done, you know.”*

*Mental Health, Not a Priority* – The majority of the participants felt that mental health was not given priority in school and workplaces, which made early prevention and treatment difficult. In the workplaces, they felt that employers found it easier to promote physical health. They also felt that employers found it difficult to talk about the role of stress and its association with mental health conditions in some industries where stress was high. They felt stress is evitable given the nature of the job; employers may therefore not want to point out an association between stress and mental disorders. With regard to educational settings, the majority of the participants felt that teachers and schools prioritise primarily on education and managing the academic needs of children and parents’ expectations. Insomuch that they are often not trained in any way to help someone with a mental health condition, nor would they have the time to delve into it deeply given other conflicting responsibilities. Consequently, teachers may find it challenging to recognize or help children with mental health needs.

*“Mental health is not a priority when it comes to talking about the health of employees; we sense that most companies are quite happy to do things that they can wrap their head around. So, conduct physical activity sessions, you know? Or have a healthier pantry, something like that, you know? Fruit day, you know? So, it’s easier. But when it comes to talking about mental health, it’s a bit more difficult.”*

*“What I observed as well… teachers have a lot on their plates, so it’s very hard for them to manage children with varying abilities in the class, much less to deal with identifying children who may need help with mental health issues, right?”*

*Forcing Mental Health into a ‘Health’ Box* – Policymakers/advisors unanimously felt that mental illnesses were seen solely as a health concern. In reality, social determinants played a significant role in the prevention and treatment of mental illnesses. On the other hand, they also highlighted groups that may be perceived to be at ‘high-risk’ of abuse or criminal offending were often not screened for mental health conditions. Such artificial silos led to a lack of holistic care of the person with mental health conditions.

*“And I think there has always been that focus on the medical approach to mental health. Where I think I want to come in is the non-medical approach, which is what I’m telling other ministries. It’s not something to ignore, but I think these other ministries may not see it. And it’s definitely not a whole-of-government awareness yet.”*

*“Erm, relax this wall between health and social services so that we can do more work across. There can be more, yea. For example, if you consider even something as simple as step-down facilities for your teenagers from a tertiary hospital – who does it? So many discussions just “it’s not my job, it’s your job, you fund, I fund, who gets the funding, who…” Everybody doesn’t want to take responsibility.”*

## Discussion

The findings of our study revealed the perspectives of policymakers and policy advisors on the impact of mental health stigma on the development and implementation of mental health programmes, strategies, and services in Singapore. The superordinate themes included: stigma as a barrier to mental health treatment, community-level barriers to mental health recovery, and mental health as a neglected need.

The participants were knowledgeable about the significant treatment gap in Singapore (Subramaniam et al., 2020a) and identified discrimination and label avoidance as significant factors that impeded help-seeking and the rejection of professional mental health services. This finding triangulates well with the results of an earlier local study examining stigma among Singaporean youth. About half of the youth surveyed stated that they would be embarrassed if diagnosed with a mental illness. Youth in this study felt that mental illness was perceived as a mark of shame, and their peers would stigmatise them if they were diagnosed with a mental health condition (Pang et al., 2017). A qualitative study conducted among patients in the United Kingdom similarly found that participants felt stigmatized even in the absence of discrimination. On receiving the diagnosis of a mental disorder, they reported feelings of anger, fear, anxiety, isolation, guilt, embarrassment—and importantly, the inclination to avoid help-seeking (Dinos et al., 2004). Perceived costs and benefits influence the decision to seek treatment. Stigma in its various forms influences this decision. While benefits may not be apparent, costs including self-stigma and treatment stigma are perceived more acutely (Clement et al., 2015) and may lead to rejection of the diagnosis and, subsequently, healthcare services.

The relationship between culture and stigma is complex. In the current study, this emerged in the theme of the family’s role in stigma and preventing help-seeking. Cultural factors were perceived to influence family member’s understanding of mental illness and avenues of help-seeking. Participants mentioned that despite Singapore being a developed and highly literate country, many older adults still believed in supernatural causes of mental illness. They felt that this would add to the sense of shame and need for secrecy (e.g., the notion that the entire family was being punished for some transgression in the past). As a result, families would choose to keep experiences related to mental illness a secret. These beliefs would also lead to seeking help from religious or traditional healers rather than medical care providers (Tan et al., 2020). In Asian societies, families are the gatekeeper for access to care, especially for children, adolescents, and even older adults who live with their adult children. Most outreach efforts and services are targeted towards the person with a mental disorder; the involvement of families in mental health education needs more focus.

Structural stigma hinders access to high-quality mental health care, including the lack of adequate mental health benefits. Access to insurance systems remains challenging for those with mental disorders, with mental health benefits often not being offered with general health benefits (Barry et al., 2010). In Singapore, only one insurance company provides coverage for mental health conditions that develop after purchasing the policy (AIA, 2020). Until recently, most job applicants were required to declare mental health conditions in their job applications. While these employers intended to cater to the needs of their employees, many people with mental disorders felt that it resulted in their being excluded from job interviews and even the consideration by employers (Subramaniam et al., 2020b). In January 2020 (after the interviews were completed), the Tripartite Alliance for Fair and Progressive Employment Practices (TAFEP) updated its guidelines to indicate that job applicants need not declare their personal information such as their mental health condition unless it is a job-related requirement. TAFEP also stated that employers should remove all declarations on mental health conditions from the job application forms (Tee, 2020).

The need to enhance and expand community-based services was mentioned by most of the participants. The debate on community versus hospital-based psychiatric care remains ongoing across most countries. A systematic review concluded that rather than adopting a hospital-only or community services alone approach, there is a need for “balanced care” whereby front-line services are based in the community, with hospitals providing acute in-patient care when necessary (Thornicroft & Tansella, 2003, 2004). In Singapore, mental health services were traditionally provided by a tertiary psychiatric institution, and over the past decade, several initiatives have been launched to enhance community care. The Ministry of Health of Singapore implemented the National Mental Health Blueprint in 2007, followed by the launch of the Community Mental Health Masterplan in 2012. This included setting up the Community Resource, Engagement and Support Team (CREST)—a community outreach team, and the Community Intervention Team (COMIT)- a multidisciplinary allied health team to enhance community care (Ong, 2017). However, the development and delivery of evidence-based services at the community level requires the wider involvement of other stakeholders. The Ministry of Health acknowledged this need and advocated for ‘adopting a whole-of-government approach to review the overall strategy on mental health together with other ministries and stakeholders’ in 2020 (Ministry of Health, 2020).

Home-based services and therapy are resource-intensive and may be limited in terms of sessions and involvement of a multidisciplinary team because of the costs incurred, not to mention other considerations such as confidentiality and privacy, boundaries, and competency issues which also pose challenges. Be that as it may, it is still an avenue that is worth exploring further, as reviews have demonstrated the efficacy of these services, and they should be considered for a sub-group of patients who face challenges in traveling (Klug et al., 2019). Tele-mental health care is another viable alternative that hospitals could invest in to provide effective care while reducing barriers to accessibility (Langarizadeh et al., 2017).

Other than funding, participants also talked of competing priorities and the lack of importance placed on mental health. Both at workplaces and schools, participants felt that mental health was not prioritized. Policymakers/advisors identified the challenges of mental health services (education, early detection, and referral) provided by academic staff whose training and experience may be limited. While this expertise would help them manage the complex school culture and educational needs of children, they would also need to prioritize academic targets. Thus, they may work competently with students with educational difficulties resulting from emotional and behavioural issues but may not have the training to identify the underlying psychiatric problem or discuss it with the child or their parents (Fazel et al., 2014). While services like REACH (Response, Early intervention, and Assessment in Community mental Health) have been set up in Singapore to work closely with schools, to help students with emotional, and behavioural issues (Lim et al., 2017), teachers remain both the first point of contact and the person with the most contact with students.

Policymakers and advisors similarly felt that while companies are engaged in wellbeing activities (mental health awareness week etc.), these efforts are often not part of a long-term strategic priority but more ad-hoc in nature. A study conducted by Deloitte in the United Kingdom reported that only 22 percent of line managers had received some form of training on mental health at work, even though 49 percent said that even basic training would be helpful. In addition, 86 percent of employees stated that they would think twice before offering help to a colleague whose mental health concerned them. Thus, while employers may understand that employees’ mental health is important, it does not always translate into taking actions to improve it (Deloitte, 2017). Currently, there are no studies in Singapore that examine the mental health needs of employees. In their article on mental health in workplaces, Pfeffer and Williams (2020) outline a clear strategy to improve workplace mental health. The process includes measuring the extent of the problem, making mental health a priority in workplaces, holding leaders accountable for employee mental health, exploring new solutions including online interventions, ensuring behavioral health coverage, and considering onsite mental health services.

Participants acknowledged the complex nature of mental disorders and the role of social determinants and social networks in developing and recovering from mental disorders. However, they felt that the provision of care continued to be fragmented across health and social sectors. Social services providers, who are traditionally disconnected from the health system, play an essential role in providing services for these patients. Innovative programs that connect healthcare providers with community-based organizations addressing social needs are being initiated in the United States. Such programs leverage innovative payment and service delivery and result from both top-down reforms initiated by federal policy and bottom-up projects driven by communities (Amarasingham et al., 2018). While similar initiatives have been launched in Singapore, evidence of the uptake and successful implementation of such programmes and their outcomes are limited (Ministry of Health, 2018). Funding remains a major source of contention as each sector is funded separately, and every sector has its workflow and care protocols. Data sharing and risk sharing are also fraught with challenges. There is thus a need for specific policies and solutions that address some of these challenges to ensure seamless care and good outcomes, especially for patients with complex needs.

### Limitations of the Study

This study included a small sample of participants. Future studies should include more participants who represent diverse ministries and roles. This might allow for a broader understanding of the barriers associated with mental health policy development and implementation. While participants were assured of confidentiality and all interviews were conducted by trained senior staff, it is possible that they were not completely candid in their discussions. It is also possible that some of the views expressed by the participants were coloured by social desirability. Participants may have felt that they were representing their organization and withheld their personal views. The participants did not include many elected officials who could have an important influence in shaping the mental health landscape of the country in terms of destigmatizing mental illnesses, advocating for those with mental illness, and on the allocation of resources as well as on the formulation and implementation of policies. We do acknowledge this as a limitation of our study as elected officials may have very different views (Purtle et al., 2017). The researchers also perceived that since participation was voluntary, many who chose to participate in the study advocated strongly for mental health. Thus the views of those who may have negative attitudes are probably not reflected in this study.

### Conclusion

Policymakers/advisors demonstrated an in-depth and nuanced understanding of the barriers (consequent to stigma) to mental healthcare delivery and access. While the themes related to public and self-stigma were similar to that expressed by the general public, these policymakers/advisors were able to associate it with help-seeking barriers based on personal experiences, knowledge, and insight gained through the implementation of mental health programmes and initiatives. Understanding and engaging policymakers is often challenging for researchers (Ion et al., 2019; Whitty, 2015). Collaboration between researchers and policy makers will lead to mutual learning and a deeper understanding of the nature (and limitations) of research and policy formulation. A better appreciation of the relevance of each other’s expertise will pave the way for evidence-based mental health policies (Kok et al., 2016; Williamson et al., 2019). The study highlights the need for policies to cut across sectors beyond health and the importance of engaging and collaborating with the wider community to overcome the challenges. A whole of society approach is required for the promotion and destigmatization of mental health. It should involve not only government agencies but also members of the public, the private sector, and advocacy groups. Such collaborations would also advocate, initiate, and implement various interventions and programmes to address the barriers to care and make for a more equitable health care system.

## Supplementary Information

Below is the link to the electronic supplementary material.

Supplementary file1 (DOCX 17 kb)
