# Supplementary material for: A Qualitative Exploration of the Views of Policymakers and Policy Advisors on the Impact of Mental Health Stigma on the Development and Implementation of Mental Health Policy in Singapore
Source: Adm Policy Ment Health. 2021 Sep 29;49(3):404–14. doi: 10.1007/s10488-021-01171-1 (PMC9005417; doi:10.1007/s10488-021-01171-1)
Supplement: Supplementary file 1 — Supplementary file1 (DOCX 17 kb) [file 10488_2021_1171_MOESM1_ESM.docx]

**Interview Guide for Policy Makers**

- Can you tell us in your own words what does ‘stigma’ mean to you?
- Can you give us examples of stigmatizing behaviour?
- Do you think people in general would have any negative perceptions towards persons with mental illness? Can you describe some of these negative perceptions they might have?
- What do you think could be some of the causes for stigma towards people with mental illness to arise in Singapore?
- (If needed )Why do you think some people may hold stigmatizing views towards mental illness?
- (If needed) Do you think employers may have negative perceptions towards those with mental illness?
- Some people believe that culture plays a role in stigma. What are your thoughts? If so how does it impact stigma?
- How do you think stigma affects people with mental illness?
- Does stigma play any role (positive or negative) in the planning of delivery of mental health services today?
- Can you tell us about any policies that you may have heard of in other countries that plays a role in reducing stigma towards those with mental illness.
- Are you aware of a successful campaign in any area that has reduced the stigma towards a highly stigmatised group in Singapore?
- Can you describe what you think are some of the ways/strategies that can be used to reduce stigma towards people with mental illness in Singapore?
- Are you aware of the term structural stigma? What does this term mean to you?
